# A microfluidics assay to study invasion of human placental trophoblast cells

**DOI:** 10.1098/rsif.2017.0131

**Published:** 2017-05-31

**Authors:** Yassen Abbas, Carolin Melati Oefner, William J. Polacheck, Lucy Gardner, Lydia Farrell, Andrew Sharkey, Roger Kamm, Ashley Moffett, Michelle L. Oyen

**Affiliations:** 1 The Nanoscience Centre, Department of Engineering, University of Cambridge, Cambridge CB3 0FF, UK; 2 Department of Pathology, University of Cambridge, Cambridge CB2 1QP, UK; 3 Centre for Trophoblast Research (CTR), Department of Physiology, Development and Neuroscience, University of Cambridge, Cambridge CB2 3EG, UK; 4 Department of Mechanical Engineering, Cambridge, MA 02139, USA; 5 Department of Biological Engineering, Massachusetts Institute of Technology, Cambridge, MA 02139, USA

**Keywords:** microfluidics, placentation, trophoblast, human

## Abstract

Pre-eclampsia, fetal growth restriction and stillbirth are major pregnancy disorders throughout the world. The underlying pathogenesis of these diseases is defective placentation characterized by inadequate invasion of extravillous placental trophoblast cells into the uterine arteries. How trophoblast invasion is controlled remains an unanswered question but is influenced by maternal uterine immune cells called decidual natural killer cells. Here, we describe an *in vitro* microfluidic invasion assay to study the migration of primary human trophoblast cells. Each experiment can be performed with a small number of cells making it possible to conduct research on human samples despite the challenges of isolating primary trophoblast cells. Cells are exposed to a chemical gradient and tracked in a three-dimensional microenvironment using real-time high-resolution imaging, so that dynamic readouts on cell migration such as directionality, motility and velocity are obtained. The microfluidic system was validated using isolated trophoblast and a gradient of granulocyte-macrophage colony-stimulating factor, a cytokine produced by activated decidual natural killer cells. This microfluidic model provides detailed analysis of the dynamics of trophoblast migration compared to previous assays and can be modified in future to study *in vitro* how human trophoblast behaves during placentation.

## 1. Introduction

**Figure 1.**
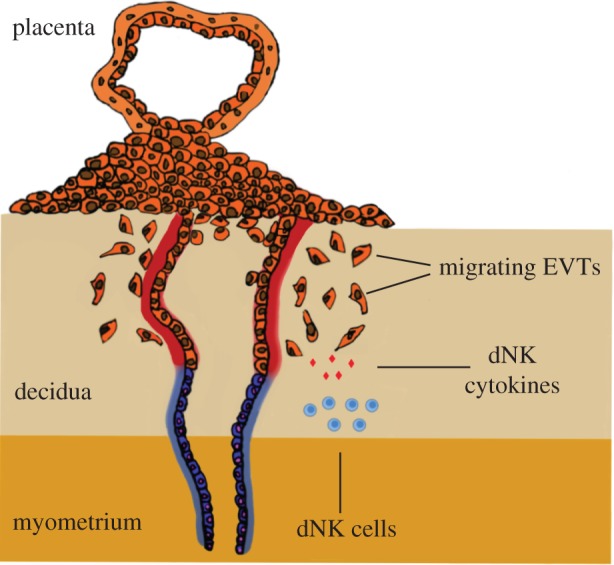
Trophoblast invasion. The placenta implants into the maternal decidua during the first trimester of pregnancy*.* Fetal extravillous trophoblasts (EVTs) detach from the implanting placenta and invade the maternal decidua to remodel uterine spiral arteries. Maternal leucocytes present at the maternal–fetal interface, including decidual natural killer (dNK) cells, may regulate trophoblast invasion and transformation of the spiral arteries by secreting cytokines such as GM-CSF. (Online version in colour.)

During the first trimester of pregnancy in humans, the process of placentation involves cells derived from the placenta, fetal extravillous trophoblasts (EVTs), invading into the uterine wall in a controlled and directed manner. Here they remodel the spiral arteries and convert them into highly dilated vessels capable of providing sufficient nutrients and oxygen to the fetus (figure 1) [1]. Insufficient trophoblast invasion leads to deficient artery remodelling and is the underlying cause of severe pregnancy disorders such as pre-eclampsia, stillbirth, fetal growth restriction and recurrent miscarriage [2,3]. A complex network of cell signalling pathways including cytokines, oxygen tension and cell–cell interactions regulate trophoblast invasion and placentation [4,5]. In addition, distinctive maternal immune cells are only present in the uterine mucosal lining, the decidua, during placentation. When these immune cells, known as decidual natural killer (dNK) cells, are activated, they produce cytokines such as granulocyte–macrophage colony-stimulating factor (GM-CSF) that increase trophoblast migration. Therefore, to understand normal and disordered pregnancy requires an understanding of how maternal immune cells and the factors they secrete regulate trophoblast invasion and thus placentation.

Conventional methods to study trophoblast invasion both *in vivo* and *in vitro* have significant drawbacks. There are marked differences in the placentation of laboratory animals when compared to humans, with the deep interstitial invasion characteristic of humans only found in the great apes [1]. *Ex vivo* explants of placentas suffer from poor viability and difficulty in sampling across the whole placenta [6]. Existing *in vitro* methods include the Transwell^®^ assay (Corning, Corning, NY, USA) where cells are placed in an insert and migrate through a cell permeable membrane towards a chemoattractant [7]. Alternatively, in the scratch assay a gap is created by ‘scratching’ a monolayer of cells and the migration rate determined by time lapse microscopy [8]. These *in vitro* assays are difficult to use with primary cells because large numbers of purified trophoblast cells from first trimester placentas are needed. Although cell lines (choriocarcinoma cell lines JEG-3 and JAR) have been used in migration assays [9–11], the expression profiles of these malignant cells are quite different from primary EVTs [12]. Moreover, these *in vitro* assays are not a measure of true chemotaxis, analysis of cell migration in two dimensions is too simplified and as such they are considered to have low physiological relevance [13,14].

In contrast to these existing migration assays, microfluidic devices allow the precise control of chemical gradients in a three-dimensional (3D) environment [15]. Cells are embedded in a physiologically relevant hydrogel matrix, and single cell chemotaxis is observed in real time under constant fluid flow [16]. Individual cell migration tracks can be quantified, and additional migration characteristics such as cell speed and directionality can be obtained [17]. Importantly, because only a few thousand cells are required, this assay can be performed using primary trophoblast cells.

Here, we describe a microfluidic device to study the directed migration of primary human trophoblast cells *in vitro.* The device was adapted from an assay to study fibrosarcoma cancer cell migration [18], since trophoblast and malignant cells share the characteristics of invasion [19,20]. The device is composed of three channels, the central one containing primary EVTs embedded in a hydrogel matrix, with two flow through channels for delivery of medium to either side of the gel. This method is validated here using the response of EVTs to GM-CSF, to demonstrate directed migration of primary trophoblast cells in a three-dimensional environment.

## 2. Material and methods

### 2.1. Fabrication of microfluidic device

**Figure 2.**
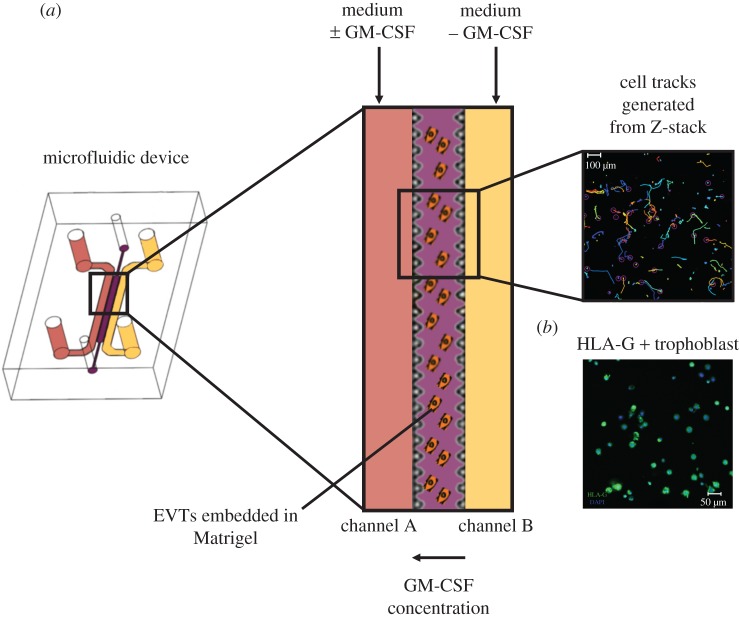
Microfluidics as a model for trophoblast invasion. EVTs are isolated from first trimester placentas, stained with a cell tracker and embedded in growth factor-reduced Matrigel in the central hydrogel channel. (*a*) A constant flow of medium is applied in the two side channels, one with (channel A) and without hrGM-CSF (channel B) to create a gradient of the cytokine across the hydrogel channel. Individual cell tracks are generated from time lapse microscopy. (*b*) To confirm the purity of cells embedded in the microfluidic device, EVT were immune-stained for HLA-G (green) and the nucleus of each cell stained with DAPI dye. (Online version in colour.)

Microfluidic devices were fabricated using soft lithography as previously described [16]. The dimensions of each device are 4.5 × 2.3 cm with the length, width and height of each channel of 20 300 µm, 1300 µm and 150 µm respectively. Ports are used to access each channel and are made using a biopsy punch. Fluid is withdrawn via channels A and B from two separate reservoirs using a syringe pump (figure 2*a*).

Microfluidic devices were filled with EVTs in ice cold (0°C) Matrigel^®^ (Corning, Corning, NY, USA). This hydrogel, a basement membrane matrix was chosen as it contains collagen IV, a protein shown to be present in the decidua and known to influence the invasive behaviour of trophoblast cells [21]. The gel was allowed to polymerize at 37°C for 45 min and then left overnight at 37°C before the start of an experiment. Experiments were carried out at a constant flow rate of 50 µl h^−1^ for 12 h.

### 2.2. Imaging and gradient validation

**Figure 3.**
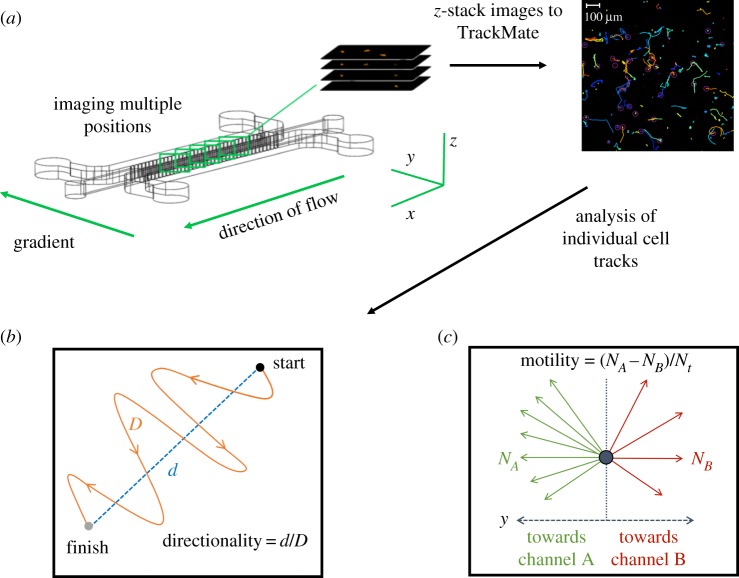
Image acquisition and analysis of individual cell tracks. (*a*) Primary EVTs embedded in Matrigel are subjected to a chemical gradient of hrGM-CSF for 12 h. EVTs are tagged with a fluorescent cell tracker and imaged at five positions using an LSM 700 confocal microscope in the *x*, *y* and *z*-direction. Images are processed with TrackMate (FIJI) and converted into cells tracks and then quantified into velocity, directionality and motility. The track colours are automatically generated by TrackMate and have no significance. (*b*) Directionality is the ratio of the net distance a cell migrates over the total distance. (*c*) Motility is found by subtracting the number of cells migrating upstream towards channel A (*N_A_*) by the number of cells migrating downstream (*N_B_*) away and dividing this by the total number of cells (*N_t_*). (Online version in colour.)

For live-cell imaging and gradient checks, the LSM700 confocal laser scanning microscope (Zeiss Oberkochen, Germany) including the Zen software was used. The device and tubing were maintained at 37°C during imaging. For cell tracking, a scanning range of 120 µm was chosen, ensuring that cells attached to the bottom coverslip of the device were excluded. Z-stacks at five positions in the central hydrogel area were acquired at 555 nm every 20 min for a total of 12 h (figure 3*a*). For chemical gradient validation identical positions and *z*-stacks were acquired at 488 nm. Fluorescein-conjugated dextran (40 kDa) at a flow rate of 50 µl h^−1^ was added to one of the two medium channels and images taken over 12 h to visualize gradient generation.

### 2.3. Analysis

To automatically track cells, the image processing software Fiji (ImageJ, v. 2.0.0-rc-14/1.49 g) and the built-in plug-in TrackMate (version 2.5.0) were used. To further analyse and interpret the data a Matlab (Mathworks, Natick, MA, USA) script was developed that could quantify cell velocity, directionality and motility. Directionality is given as the ratio of the net distance a cell moved to the total distance migrated (figure 3*b*) [22]. Quantifying the fraction of cells migrating downstream and subtracting these from the fraction of cells migrating upstream determined motility (figure 3*c*).

### 2.4. Computational model

A finite-element model (FEM) was developed in Comsol Multiphysics (COMSOL, Stockholm, Sweden) by importing the device geometry from Auto-CAD (Autodesk, San Rafael, CA, USA). The model was implemented to determine the molecular gradient by solving the time-dependent diffusion equation:
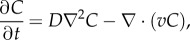
where *C* is the concentration, *t* is time, *D* is the diffusivity of the solute, and *v* is the fluid velocity. The model solved the diffusion equation for the full three-dimensional geometry of the microfluidic device. The diffusivity was defined as 2 × 10^−11^ m^2^ s^−1^ [23] and assumed to be constant throughout the hydrogel region. The inlet concentration of the source channel and the inlet flow rate were defined by the experimental values of 0.2 mol m^−3^ and 1.4 × 10^−4^ m s^−1^, respectively, and no-flux and no-slip boundary conditions were applied at the PDMS walls.

### 2.5. Isolation of primary cells and cell seeding

Trophoblast and decidual leucocytes (DLs) were isolated from placental and decidual samples from normal pregnancies between 7 and 12 weeks of gestation using published protocols [12,24]. Ethical approval was obtained from Cambridge Local Research Ethics Committee (reference no. 04/Q0108/23; Cambridge; United Kingdom).

Primary isolates of trophoblast cells were cultured in Fluorobrite DMEM medium (Thermo Fisher Scientific, Waltham, MA, USA), supplemented with 20% fetal calf serum (FCS, Biosera, Nuaille, France), 1 mM sodium pyruvate (Thermo Fisher Scientific, Waltham, MA, USA), 1× MEM non-essential amino acids, 2 mM l^−1^ glutamine, 10 units ml^−1^ penicillin, 100 µg ml^−1^ streptomycin and 2 mg ml^−1^ gentamycin (Thermo Fisher Scientific, Waltham, MA, USA). Following overnight culture on fibronectin-coated wells, the cultures typically yield approximately 70–90% EVTs identified by flow cytometry using an antibody specific for HLA-G [24]. HLA-G is a HLA class I molecule uniquely expressed by invasive EVTs and is used to check purity of the isolated first trimester cells. Trophoblast cells were labelled by incubation with 40 µM cell tracker orange CMTMR (Thermo Fisher Scientific, Waltham, MA, USA) for 15 min at 37°C. EVTs at a concentration of 8 × 10^6^ cells ml^−1^ in cell medium were then embedded in a 1 : 1 mixture of Matrigel:cell solution. The volume of gel in the central channel is approximately 2 µl with approximately 2000 cells seeded. To generate a gradient of human recombinant (hr) GM-CSF, 10 ng ml^−1^ was added to the medium in channel A. Cells were tracked in three dimensions by taking images every 20 min for 12 h.

The dNK cells stochastically express polymorphic receptors (Killer-cell Immunoglobulin-like receptors, *KIR*) that can impart either an activating or inhibitory signal to the cell. The dNK cells from different donors were genotyped to determine if they possessed one of these *KIR*, the activating *KIR2DS1* gene. Briefly, genomic DNA was isolated from decidual samples using the QIAamp DNA Mini Kit (QIAGEN, Hilden, Germany). The presence or absence of *KIR2DS1* in each decidual sample was determined by genotyping *KIR* from genomic DNA by PCR as described previously [25,26]. dNK cells were isolated from first trimester decidual samples of *KIR2DS1+* and *KIR2DS1−* donors. The isolates were cultured overnight in RPMI1640 (Thermo Fisher Scientific, Waltham, MA, USA), antibiotics, 10% FCS and 2.5 ng ml^−1^ Interleukin 15 (IL-15) (Peprotech, Rocky Hill, NJ, USA). Enrichment for dNK cells was performed using the EasySep™ Human NK Cell Enrichment Kit (STEMCELL Technologies, Vancouver, Canada), which uses magnetic beads to select for CD56+ cells. The purity of dNK cells was established by staining for CD56-PE (clone HCD56, Biolegend, San Diego, CA, USA) using flow cytometry as described previously [27]. After enrichment of DL, the purity of dNK cells increased from 48% to 85%.

### 2.6. Immunofluorescence staining and quantification of HLA-G+ trophoblast cells

At the end of each experiment, trophoblast cells were identified within the device by staining for HLA-G to determine purity (figure 2*b*). The devices were washed with 1× PBS and incubated with 4% paraformaldehyde (Sigma, St. Louis, MI, USA) for 30 min. The devices were blocked with 1% PBS and 2% normal goat serum (Sigma, St. Louis, MI, USA) for 2 h followed by the incubation with anti-HLA-G (clone G233-216, Quantum Biosystems, Osaka, Japan) in 1× PBS at 4°C overnight. The device was washed twice with 1× PBS. FITC labelled Anti-HLA-G (MEMG9-FITC, Bio-RAD, Kidlington, UK) and the nuclear counterstain DAPI (Sigma, St Louis, MI, USA) were then applied for 2 h. Four representative images across the hydrogel channel after each experiment were acquired using a Zeiss LSM 700 (Zeiss, Oberkochen, Germany) confocal microscope. The percentage of HLA-G+ trophoblast cells was determined by counting the total number of nuclei and the number of HLA-G+ cells. The average purity of isolated EVTs was 80% over five experiments, in accordance with previous findings using flow cytometry [28].

### 2.7. Activation of decidual natural killer cells

To activate dNK cells, KIR receptors were cross-linked using plate-bound antibodies. dNK cells from *KIR2DS1*+ donors were isolated and used after overnight culture to remove adherent cells. Eight-well strips (Sigma, St Louis, MI, USA) were coated with 200 µl per well of 2.5 mg ml^−1^ monoclonal antibody (mAb), EB6 (CD158a, with specificity for KIR2DS1/KIR2DL1, Beckman Coulter, High Wycombe, UK) or control human IgG (Biolegend, San Diego, CA, USA) in 10 mM HEPES (PAA) overnight at 4°C. The dNK cells were plated at 2 × 10^5^ cells per well in RPMI1640, antibiotics, 10% FCS and 2.5 ng ml^−1^ IL-15 and cultured at 37°C for 24 h. The supernatant was then collected and GM-CSF was quantified using DuoSet ELISA (R&D Systems, Minneapolis, MN, USA) according to the manufacturer's instructions. The dNK supernatants obtained following activation of KIR2DS1+ dNK cells were diluted and used at a GM-CSF concentration of 15 pg ml^−1^.

### 2.8. Statistical analysis

Cell migration characteristics were analysed using Prism Software (Graphpad, La Jolla, CA, USA). The unpaired *t*-test and one-way analysis of variance (ANOVA) were used for the hrGM-CSF and dNK supernatant experiments, respectively. Significance is set as *p* < 0.05.

## 3. Results

### 3.1. Establishing gradient in the microfluidic device

**Figure 4.**
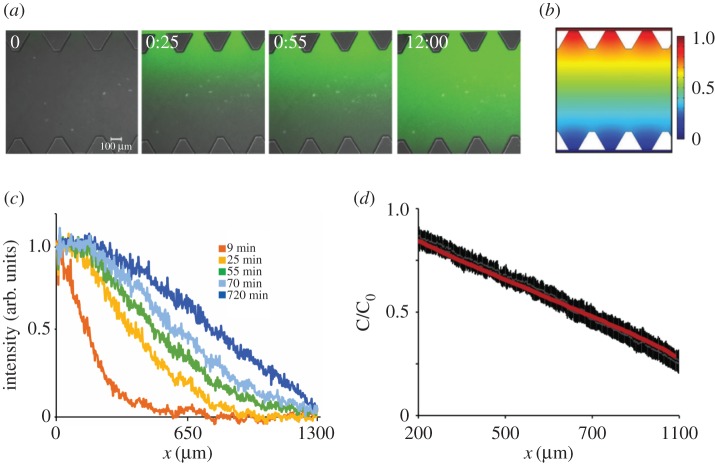
Characterization of the chemical gradient across the hydrogel channel during constant fluid flow. (*a*) Fluorescein dextran of similar molecular weight to human GM-CSF was added to the medium in channel A, constant fluid flow of 50 µl h^−1^ per channel was applied, and the gradient was investigated for 12 h. The gradient gradually builds up over a time period of 1 h and is stable for at least 12 h. (*b*) Computational model for gradient generation demonstrates similar gradient at the device mid-plane at steady state to experimentally observed gradient 12 h. (*c*) Plot of fluorescein intensity across device, with width 1300 µm demonstrates that linear gradient is established after 1 h. (*d*) Plot of mean intensity and standard deviation (black bars) for four independent devices demonstrates linear gradient in the central hydrogel region, with width 900 µm (each triangular post is 200 µm in length) is similar to the computationally predicted gradient (red line). C/C_0_ is concentration of tracer normalized by the concentration in the media channel. (Online version in colour.)

The chemical gradient between the two medium delivery channels and the central channel containing cells was characterized to determine whether a stable gradient was generated and how long this gradient remained. The gradient gradually built up until stabilizing after approximately 1 h and was steady for at least 12 h (figure 4*a*). The FEM implemented in COMSOL was used to characterize transport phenomena in the device that corresponds to the 40 kDa dextran diffusing inside the matrix (figure 4*b*). The computational model confirms the gradient profile is similar to the one observed experimentally at 12 h with a linear gradient gradually building up after 1 h as shown by a plot of fluorescein intensity measurements across the device at different time points (figure 4*c*). To evaluate whether experimental and computationally predicted gradients are similar, the mean intensity profiles of four independent devices were compared to the gradient predicted by the FEM (figure 4*d*). The overlay demonstrates that linear gradients during microfluidic experiments (black) are similar to the computationally predicted gradient (red). C/C_0_ is concentration of tracer normalized by the concentration in the media channel.

### 3.2. Trophoblast cells in granulocyte-macrophage colony-stimulating factor gradients

**Figure 5.**
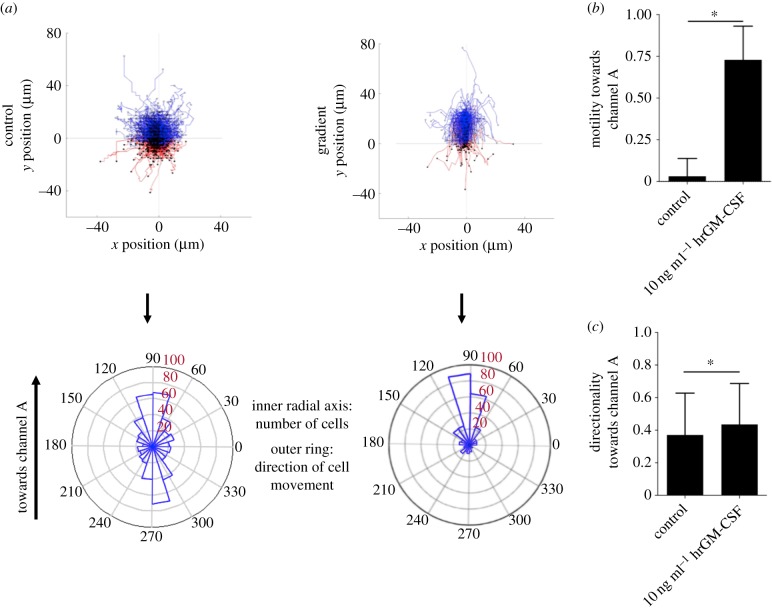
Recombinant hrGM-CSF stimulates the migration of human extravillous trophoblasts (EVTs). (*a*) Cell tracks and polar histograms show the random migration of EVTs in the control compared to directed migration when 10 ng ml^−1^ GM-CSF is added to channel A. A representative experiment is shown out of five performed. Only cells that migrated more than 30 µm were analysed. Cells migrating towards channel A (hrGM-CSF) and towards channel B (no hrGM-CSF) are coloured blue and red, respectively. Polar histograms visualize the number of cells that migrate in different directions (inner radial axis). The outer radial axis represents migration angles. (*b*) The difference of cell fractions migrating towards and away from hrGM-CSF was quantified. Significantly more cells migrate towards GM-CSF when exposed to 10 ng ml^−1^ hrGM-CSF (*n* = 5). (*c*) Trophoblast cells migrate with increased directionality in the presence of GM-CSF. *p < 0.05 calculated using the unpaired *t*-test. (Online version in colour.)

Recombinant GM-CSF, a product of activated dNK cells, has previously been shown to enhance trophoblast invasion using the Transwell assay^®^ [29] and was used here to validate migration of primary trophoblast cells in the microfluidic device. Figure 5*a* shows plots of single cell migration tracks (top) and the net direction of cells in polar histograms (bottom) for control (no gradient) and hrGM-CSF gradients, respectively. Data are shown for one representative device of five repeats. There is undirected migration when hrGM-CSF is absent. In contrast, hrGM-CSF induced a clear increase in migration towards the channel with hrGM-CSF present.

The average migration velocity of EVTs with no gradient over five samples is 6.52 ± 0.91 µm h^−1^. When the hrGM-CSF gradient is present, the average migration velocity significantly decreases to 6.26 ± 0.74 µm h^−1^ (electronic supplementary material, figure S1) and trophoblast cells move with significantly increased directionality, from 0.37 to 0.44 (figure 5*c*).

For each experiment, migration towards and away from the concentration source was quantified. When hrGM-CSF is added, a significantly larger fraction of EVTs migrates towards this stimulus compared to the control, from 0.04 to 0.73 (figure 5*b*). Thus, the hrGM-CSF gradient substantially enhances the migratory behaviour of trophoblast cells.

### 3.3. Activation of decidual natural killer cells induces trophoblast migration

**Figure 6.**
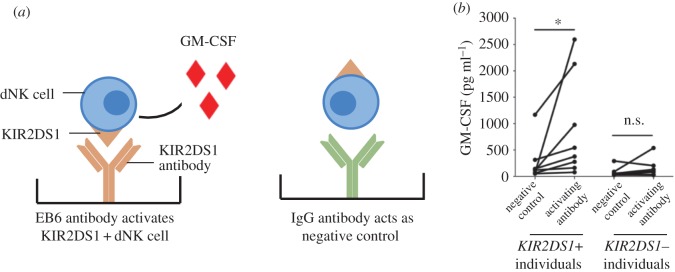
GM-CSF produced by dNK cells after activation of KIR2DS1. To activate KIR2DS1, dNK cells are stimulated with plate-bound (*a*) mAb, EB6 or (*b*) negative control IgG. The levels of GM-CSF are measured by ELISA and show a significantly increased production of GM-CSF by dNK cells from donors with the *KIR2DS1* gene but not those who lack it. *p < 0.05, ns—not significant, determined using the Wilcoxon rank-sum test. (Online version in colour.)

We next asked whether cytokines and chemokines produced following activation of dNK cells have the same positive effect on trophoblast migration. The activating NK receptor, *KIR2DS1*, is present in approximately 45% of donors. Donors were typed for the presence/absence of the gene and dNK cells expressing KIR2DS1 were activated using a specific mAb (figure 6*a*) (§2.7). Similar to previous results [29], GM-CSF production increased in the supernatants from *KIR2DS1+* but not *KIR2DS1−* donors compared to the control (*n* = 8) (figure 6*b*).

**Figure 7.**
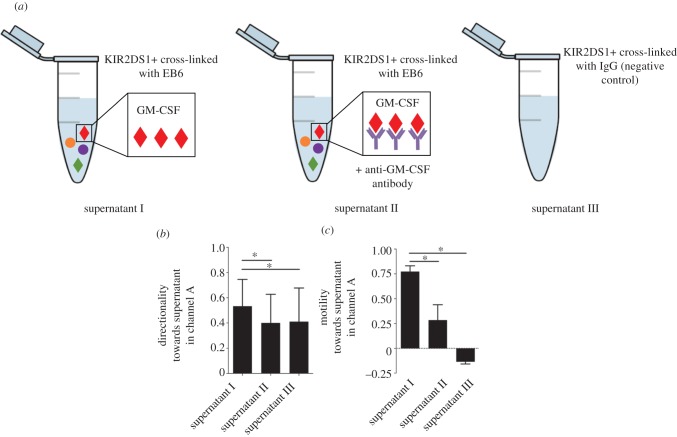
GM-CSF produced by dNK cells after activation of KIR2DS1 stimulates trophoblast migration. Trophoblast migration measured with a chemical gradient of supernatants from dNK cells after (*a*) KIR2DS1+ dNK cells activated by mAb, EB6 (supernatant I), anti-GM-CSF neutralizing antibody (supernatant II) or negative control (supernatant III). (*b*) Trophoblast cells migrate with increased directionality towards supernatants produced by activated KIR2DS1+ dNK cells. Directed migration is reduced when anti-GM-CSF neutralizing antibody is added. (*c*) A large fraction of cells migrate towards increasing concentrations of supernatants produced by activated KIR2DS1+ dNK cells. *p < 0.05 calculated using one-way analysis of variance (ANOVA) and Dunn's multiple comparisons test. (Online version in colour.)

The dNK supernatants from *KIR2DS1+* donors were added to channel A to generate a gradient (figure 7*a* supernatant I). In parallel, a GM-CSF neutralizing antibody was added with the KIR2DS1+ supernatant to test whether GM-CSF is the cytokine produced by activated dNK cells that is causing trophoblast cells to migrate (figure 7*a* supernatant II). In addition, supernatant from *KIR2DS1+* donors cross-linked with IgG control antibody was added to channel A (figure 7*a* supernatant III). Trophoblast cells preferentially migrated with increased directionality (figure 7*b*) and motility (figure 7*c*) towards the supernatants obtained from activating dNK cells compared to random migration in the supernatants with negative control containing IgG antibody (*n* = 5). Increase in directionality was diminished with the addition of the GM-CSF neutralizing antibody, whereas the motility decreased but not to the same level of the negative control. Thus, activation of maternal dNK cells does result in increased trophoblast migration and this is partially due to GM-CSF.

## 4. Discussion

In this study, microfluidic devices have been used to quantify the migratory characteristics of primary trophoblast cells to model EVT migration, which is a critical period in human pregnancy. In previous work [29] GM-CSF was shown to be a chemoattractant for EVTs; this new approach allows for accurate quantification of migration of individual cells in response to the chemoattractant. In addition, activation of dNK cells through an NK receptor, KIR2DS1, results in GM-CSF secretion that enhances the directional migration of trophoblast cells. This illustrates how maternal uterine immune cells can affect placentation, and how this microfluidics assay approach can be used for biological studies of trophoblast migration.

Although invasion of the uterus by trophoblast cells plays a critical role in reproductive outcome, limited understanding of the mechanisms controlling trophoblast invasion remains a major obstacle to progress in pregnancy research [20,30]. Learning more about what factors influence trophoblast invasion and the cellular and molecular basis of the invasion pathway is essential. Reliable and reproducible methods to study how trophoblast behaviour is controlled, and that can be used in different laboratories with primary cells is lacking. Strategies to improve methods to study cell migration have come from the cancer field, since cancer cells spread through tissues [31–33]. Microfluidics has been used to study various aspects of tumour biology such as intravasation [18], extravasation [34], angiogenesis [35], tumour–stroma interactions [36], tumour cell migration in response to chemotaxis [37], matrix stiffness [38] and interstitial flow [39]. In addition, candidate molecules and drugs to target cancer metastasis can be tested using microfluidic devices because of the low cost of reagents and manufacturing the devices, together with the high throughput nature of testing [40,41].

The microfluidic device used here has been adapted to observe and quantify trophoblast. Both single cell and collective migration, together with quantification of migration speed and directionality, provide key advantages over conventional assays used to study EVTs. Each assay can be performed with a small number of cells, which makes it possible to conduct research on human samples given the challenges of isolating sufficient number of pure primary HLAG+ EVTs from first trimester placentas.

To validate migration of EVTs in the microfluidic device, a gradient of hrGM-CSF (previously shown by us to enhance trophoblast migration in a Transwell^®^ assay [29]) was generated in the microfluidic device. Using real-time, high-resolution imaging, the velocity of trophoblast migration is similar to that of invasive cancer cells [42,43]. In the absence of a chemical gradient, the movement of EVTs is undirected but with a chemical gradient of hrGM-CSF, trophoblast moves with increased directionality and motility but reduced velocity towards the GM-CSF positive control. Immunohistochemistry for HLA-G was performed after each experiment to assess the purity of EVTs in the population. In future it should be possible to retrieve cells from the central hydrogel channel for other techniques such as gene expression and flow cytometric analysis.

Having established the microfluidics platform as viable for the study of trophoblast migration, more complex biological questions can be asked. Maternal *KIR* genes are highly polymorphic and it is unclear how the presence or absence of particular KIR in different women translates into a functional effect on trophoblast cells early in gestation [25,26]. Previous genetic studies show that an activating NK receptor, *KIR2DS1*, protects women from pre-eclampsia and KIR2DS1+ dNK cells promote trophoblast cell migration *in vitro* compared to women who only express the inhibitory, KIR2DL1 [25,26]. The results in this study are consistent with this model. The activation of KIR2DS1+ dNK cells resulted in significantly higher secretion of GM-CSF than KIRDS1− dNK cells when compared to the negative IgG controls [29]. A larger fraction of trophoblast cells migrated towards supernatants secreted from KIR2DS1+ dNK cells with increased directionality and motility compared to random migration in the control. To determine if GM-CSF in the supernatant was responsible for this effect, the addition of anti-GM-CSF antibody resulted in a reduction in the directionality and motility of cells migrating towards the concentration source. Therefore, GM-CSF is a key cytokine in the regulation of trophoblast invasion by dNK cells but other factors not yet definitively identified are clearly involved as the migration was not completely blocked by the anti-GM-CSF antibody. This microfluidic migration assay provides a three-dimensional model for trophoblast invasion through the extracellular matrix (ECM) involving highly regulated and reciprocal interactions between ECM and cells. Because the migration response is governed by the ability of a cell to degrade the ECM, it is important to ask whether the ECM used in this study is truly representative of decidual matrix components. During the transformation of endometrium to decidua to prepare the uterine lining for implantation and pregnancy, there is a profound change in the ECM with a large increase in collagen IV and laminin surrounding endometrial stromal cells, which are the main protein components of Matrigel, the basement membrane used in this study [44,45]. In addition, the decidua contains collagen type I, III and VI, which are diffusively distributed in the endometrium throughout the cycle [46,47]. Moreover, the basement membrane at the end of the first trimester surrounding each decidual stromal cell contains fibronectin and heparin sulphate proteoglycan meaning that ECM components present in the decidua are more varied than in Matrigel. A second important question is whether the mechanical stiffness of the hydrogel surrounding the cells is representative of decidual tissue. The mechanical environment is known to influence cellular responses including adhesion and migration, as cells are able to convert mechanical input into complex intracellular signalling cascades and downstream protein expression [48]. However, the mechanical stiffness of decidua remains uncharacterized and there are variations in the reported stiffness of Matrigel [49,50]. Therefore, more work in future is needed to better understand both the mechanical and molecular properties of the decidua and to design a gel that better represents the tissue trophoblast cells migrate through *in vitro*.

## 5. Conclusion

This study has described a new approach to study human trophoblast invasion. By using a bioengineering approach, we have retained the physiological relevance of trophoblast cells invading in three dimensions. This was done while adding analysis of the dynamics of cell migration, which is not possible in conventional assays and animal models. This study goes further than previous work to add greater quantification on GM-CSF as a chemoattractant for EVT migration. With the use of microfluidic devices there is potential to further investigate the complex physiochemical influences on trophoblast behaviour during placentation.

## Supplementary Material

Trophoblast migration velocity

## Data accessibility

Supporting research data including code to analyse cell migration tracks may be accessed at https://doi.org/10.17863/CAM.7998.

## References

[1] Moffett A, Loke C 2006 Immunology of placentation in eutherian mammals. Nat. Rev. Immunol. 6, 584–594. (10.1038/nri1897) 16868549

[2] Brosens I, Pijnenborg R, Vercruysse L, Romero R 2011 The Great Obstetrical Syndromes are associated with disorders of deep placentation. Am. J. Obstet. Gynecol. 204, 193–201. (10.1016/j.ajog.2010.08.009) 21094932 PMC3369813

[3] Pijnenborg R, Anthony J, Davey DA, Rees A, Tiltman A, Vercruysse L, Assche AV 1991 Placental bed spiral arteries in the hypertensive disorders of pregnancy. Br. J. Obstet. Gynaecol. 98, 648–655. (10.1111/j.1471-0528.1991.tb13450.x) 1883787

[4] Whitley GSJ, Cartwright JE 2010 Cellular and molecular regulation of spiral artery remodelling: lessons from the cardiovascular field. Placenta 31, 465–474. (10.1016/j.placenta.2010.03.002) 20359743 PMC2882556

[5] Burton GJ, Jauniaux E, Charnock-Jones DS 2010 The influence of the intrauterine environment on human placental development. Int. J. Dev. Biol. 54, 303–312. (10.1387/ijdb.082764gb) 19757391

[6] Miller RK, Genbacev O, Turner MA, Aplin JD, Caniggia I, Huppertz B 2005 Human placental explants in culture: approaches and assessments. Placenta 26, 439–448. (10.1016/j.placenta.2004.10.002) 15950058

[7] Chen H-C 2005 Boyden chamber assay. Methods Mol. Biol. 294, 15–22. 15576901 10.1385/1-59259-860-9:015

[8] Liang C-C, Park AY, Guan J-L 2007 In vitro scratch assay: a convenient and inexpensive method for analysis of cell migration in vitro. Nat. Protoc. 2, 329–333. (10.1038/nprot.2007.30) 17406593

[9] Lash GE, Warren AY, Underwood S, Baker PN 2002 Vascular endothelial growth factor is a chemoattractant for trophoblast cells. Placenta 24, 549–556. (10.1053/plac.2002.0923) 12744932

[10] Zhao H, Jiang Y, Cao Q, Hou Y, Wang C 2012 Role of integrin switch and transforming growth factor Beta 3 in hypoxia-induced invasion inhibition of human extravillous trophoblast cells. Biol. Reprod. 87, 47 (10.1095/biolreprod.112.099937) 22674391

[11] Pollheimer J, Husslein P, Knöfler M 2005 Invasive trophoblasts generate regulatory collagen XVIII cleavage products. Placenta 26, S42–S45. (10.1016/j.placenta.2004.12.005) 15837066

[12] Apps R, Sharkey A, Gardner L, Male V, Trotter M, Miller N, North R, Founds S, Moffett A 2011 Genome-wide expression profile of first trimester villous and extravillous human trophoblast cells. Placenta 32, 33–43. (10.1016/j.placenta.2010.10.010) 21075446 PMC3065343

[13] Roussos ET, Condeelis JS, Patsialou A 2011 Chemotaxis in cancer. Nat. Rev. Cancer 11, 573–587. (10.1038/nrc3078) 21779009 PMC4030706

[14] Katt ME, Placone AL, Wong AD, Xu ZS, Searson PC 2016 In vitro tumor models: advantages, disadvantages, variables, and selecting the right platform. Front. Bioeng. Biotechnol. 4, 12 (10.3389/fbioe.2016.00012) 26904541 PMC4751256

[15] van Duinen V, Trietsch SJ, Joore J, Vulto P, Hankemeier T 2015 Microfluidic 3D cell culture: from tools to tissue models. Curr. Opin. Biotechnol. 35, 118–126. (10.1016/j.copbio.2015.05.002) 26094109

[16] Shin Y, Han S, Jeon JS, Yamamoto K, Zervantonakis IK, Sudo R, Kamm RD, Chung S 2012 Microfluidic assay for simultaneous culture of multiple cell types on surfaces or within hydrogels. Nat. Protoc. 7, 1247–1259. (10.1038/nprot.2012.051) 22678430 PMC4035049

[17] Polacheck WJ, Zervantonakis IK, Kamm RD 2013 Tumor cell migration in complex microenvironments. Cell. Mol. Life Sci. 70, 1335–1356. (10.1007/s00018-012-1115-1) 22926411 PMC3557537

[18] Zervantonakis IK, Hughes-Alford SK, Charest JL, Condeelis JS, Gertler FB, Kamm RD 2012 Three-dimensional microfluidic model for tumor cell intravasation and endothelial barrier function. Proc. Natl Acad. Sci. USA 109, 13 515–13 520. (10.1073/pnas.1210182109) PMC3427099 22869695

[19] Ferretti C, Bruni L, Dangles-Marie V, Pecking AP, Bellet D 2007 Molecular circuits shared by placental and cancer cells, and their implications in the proliferative, invasive and migratory capacities of trophoblasts. Hum. Reprod. Update 13, 121–141. (10.1093/humupd/dml048) 17068222

[20] Zhu J-Y, Pang Z-J, Yu Y-H 2012 Regulation of trophoblast invasion: the role of matrix metalloproteinases. Rev. Obstet. Gynecol. 5, e137–e143. 23483768 PMC3594863

[21] Oefner CM et al. 2015 Collagen type IV at the fetal-maternal interface. Placenta 36, 59–68. (10.1016/j.placenta.2014.10.012) 25465704 PMC4302218

[22] Gorelik R, Gautreau A 2014 Quantitative and unbiased analysis of directional persistence in cell migration. Nat. Protoc. 9, 1931–1943. (10.1038/nprot.2014.131) 25033209

[23] Gribbon P et al. 1998 Macromolecular diffusion of biological polymers measured by confocal fluorescence recovery after photobleaching. Biophys. J. 75, 1032–1039. (10.1016/S0006-3495(98)77592-7) 9675204 PMC1299777

[24] Male V, Gardner L, Moffett A 2012 Isolation of cells from the feto-maternal interface. Curr. Protoc. Immunol. Chapter 7, Unit 7.40.1-11 (10.1002/0471142735.im0740s97) 22470137

[25] Hiby SE, Walker JJ, O'shaughnessy KM, Redman CWG, Carrington M, Trowsdale J, Moffett A 2004 Combinations of maternal KIR and fetal HLA-C genes influence the risk of preeclampsia and reproductive success. J. Exp. Med. 200, 957–965. (10.1084/jem.20041214) 15477349 PMC2211839

[26] Hiby SE et al. 2010 Maternal activating KIRs protect against human reproductive failure mediated by fetal HLA-C2. J. Clin. Invest. 120, 4102–4110. (10.1172/JCI43998) 20972337 PMC2964995

[27] Loke YW, King A 1995 Human implantation: cell biology and immunology, 1st edn Cambridge, UK: Cambridge University Press.

[28] Male V, Trundley A, Gardner L, Northfield J, Chang C, Apps R, Moffett A 2010 Natural killer cells in human pregnancy. Methods Mol. Biol. 612, 447–463. (10.1007/978-1-60761-362-6_30) 20033659

[29] Xiong S. et al. 2013 Maternal uterine NK cell-activating receptor KIR2DS1 enhances placentation. J. Clin. Invest. 123, 4264–4272. (10.1172/JCI68991) 24091323 PMC4382274

[30] Kaufmann P, Black S, Huppertz B 2003 Endovascular trophoblast invasion: implications for the pathogenesis of intrauterine growth retardation and preeclampsia. Biol. Reprod. 69, 1–7. (10.1095/biolreprod.102.014977) 12620937

[31] Mehlen P, Puisieux A 2006 Metastasis: a question of life or death. Nat. Rev. Cancer 6, 449–458. (10.1038/nrc1886) 16723991

[32] Weber GF 2013 Why does cancer therapy lack effective anti-metastasis drugs? Cancer Lett. 328, 207–211. (10.1016/j.canlet.2012.09.025) 23059758

[33] Friedl P, Wolf K 2003 Tumour-cell invasion and migration: diversity and escape mechanisms. Nat. Rev. Cancer 3, 362–374. (10.1038/nrc1075) 12724734

[34] Jeon JS, Zervantonakis IK, Chung S, Kamm RD, Charest JL 2013 *In vitro* model of tumor cell extravasation. PLoS ONE 8, e56910 (10.1371/journal.pone.0056910) 23437268 PMC3577697

[35] Vickerman V, Kamm RD 2012 Mechanism of a flow-gated angiogenesis switch: early signaling events at cell–matrix and cell–cell junctions. Integr. Biol. 4, 863 (10.1039/c2ib00184e) PMC4090039 22673733

[36] Menon N. V, Chuah YJ, Cao B, Lim M, Kang Y 2014 A microfluidic co-culture system to monitor tumor-stromal interactions on a chip. Biomicrofluidics 8, 64118 (10.1063/1.4903762) PMC4257957 25553194

[37] Chen Y-C et al. 2015 Single-cell migration chip for chemotaxis-based microfluidic selection of heterogeneous cell populations. Sci. Rep. 5, 9980 (10.1038/srep09980) 25984707 PMC4435023

[38] Pathak A, Kumar S 2012 Independent regulation of tumor cell migration by matrix stiffness and confinement. Proc. Natl Acad. Sci. USA 109, 10 334–10 339. (10.1073/pnas.1118073109) PMC3387066 22689955

[39] Polacheck WJ, Charest JL, Kamm RD 2011 Interstitial flow influences direction of tumor cell migration through competing mechanisms. Proc. Natl Acad. Sci. USA 108, 11 115–11 120. (10.1073/pnas.1103581108) PMC3131352 21690404

[40] Kuo C-T, Chiang C-L, Chang C-H, Liu H-K, Huang G-S, Huang RY-J, Lee H, Huang C-S, Wo AM 2014 Modeling of cancer metastasis and drug resistance via biomimetic nano-cilia and microfluidics. Biomaterials 35, 1562–1571. (10.1016/j.biomaterials.2013.11.008) 24269156

[41] Huang Y, Agrawal B, Sun D, Kuo JS, Williams JC 2011 Microfluidics-based devices: new tools for studying cancer and cancer stem cell migration. Biomicrofluidics 5, 13412 (10.1063/1.3555195) 21522502 PMC3082349

[42] Niggemann B et al. 1997 Locomotory phenotypes of human tumor cell lines and T lymphocytes in a three-dimensional collagen lattice. Cancer Lett. 118, 173–180. (10.1016/s0304-3835(97)00328-5) 9459207

[43] Schwartz MP et al. 2013 A quantitative comparison of human HT-1080 fibrosarcoma cells and primary human dermal fibroblasts identifies a 3D migration mechanism with properties unique to the transformed phenotype. PLoS ONE 8, e81689 (10.1371/journal.pone.0081689) 24349113 PMC3857815

[44] Gellersen B, Brosens JJ 2014 Cyclic decidualization of the human endometrium in reproductive health and failure. Endocr. Rev. 35, 851–905. (10.1210/er.2014-1045) 25141152

[45] Hughes CS, Postovit LM, Lajoie GA 2010 Matrigel: a complex protein mixture required for optimal growth of cell culture. Proteomics 10, 1886–1890. (10.1002/pmic.200900758) 20162561

[46] Iwahashi M, Muragaki Y, Ooshima A, Yamoto M, Nakano R 1996 Alterations in distribution and composition of the extracellular matrix during decidualization of the human endometrium. J. Reprod. Fertil. 108, 147–155. (10.1530/jrf.0.1080147) 8958841

[47] Zhang H, Labouesse M 2012 Signalling through mechanical inputs—a coordinated process. J. Cell Sci. 125, 3025–3038. (10.1242/jcs.095794) 22929901

[48] Provenzano PP, Keely PJ 2011 Mechanical signaling through the cytoskeleton regulates cell proliferation by coordinated focal adhesion and Rho GTPase signaling. J. Cell Sci. 124, 1195–1205. (10.1242/jcs.067009) 21444750 PMC3065381

[49] Soofi SS, Last JA, Liliensiek SJ, Nealey PF, Murphy CJ 2009 The elastic modulus of Matrigel as determined by atomic force microscopy. J. Struct. Biol. 167, 216–219. (10.1016/j.jsb.2009.05.005) 19481153 PMC2747304

[50] Wood JA, Liliensiek SJ, Russell P, Nealey PF, Murphy CJ 2010 Biophysical cueing and vascular endothelial cell behavior. Materials 3, 1620–1639. (10.3390/ma3031620)

