# Supplementary material for: A microfluidics assay to study invasion of human placental trophoblast cells
Source: J R Soc Interface. 2017 May 31;14(130):20170131. doi: 10.1098/rsif.2017.0131 (PMC5454302; doi:10.1098/rsif.2017.0131)
Supplement: Trophoblast migration velocity [file rsif20170131supp1.pdf]

Supplementary Figures

Figure S1

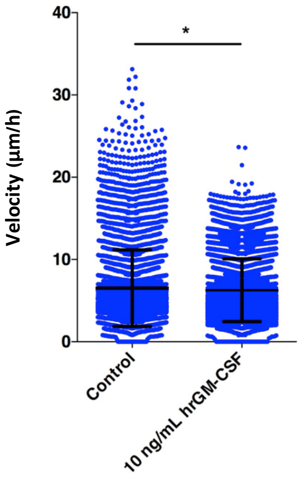

A microfluidics assay to study invasion of human placental trophoblast cells

Yassen Abbas, Carolin Melati Oefner, William J. Polacheck, Lucy Gardner, Lydia Farrell, Andrew Sharkey, Roger Kamm, Ashley Moffett and Michelle L. Oyen

Submitted to the Royal Society of Interface
